# Using a community-based definition of poverty for targeting poor households for premium subsidies in the context of a community health insurance in Burkina Faso

**DOI:** 10.1186/s12889-014-1335-4

**Published:** 2015-02-06

**Authors:** Germain Savadogo, Aurelia Souarès, Ali Sié, Divya Parmar, Gilles Bibeau, Rainer Sauerborn

**Affiliations:** Centre de Recherche en Santé de Nouna, Nouna, Burkina Faso; Institute of Public Health, University of Heidelberg, INF 324 69120 Heidelberg, Germany; School of Health Sciences, City University, London, UK; Department of Anthropology, University of Montreal, Montreal, Canada

**Keywords:** Self-assessment, Participatory, Poverty, Wealth, Community, Burkina Faso

## Abstract

**Background:**

One of the biggest challenges in subsidizing premiums of poor households for community health insurance is the identification and selection of these households. Generally, poverty assessments in developing countries are based on monetary terms. The household is regarded as poor if its income or consumption is lower than a predefined poverty cut-off. These measures fail to recognize the multi-dimensional character of poverty, ignoring community members’ perception and understanding of poverty, leaving them voiceless and powerless in the identification process. Realizing this, the steering committee of Nouna’s health insurance devised a method to involve community members to better define ‘perceived’ poverty, using this as a key element for the poor selection. The community-identified poor were then used to effectively target premium subsidies for the insurance scheme.

**Methods:**

The study was conducted in the Nouna’s Health District located in northwest Burkina Faso. Participants in each village were selected to take part in focus-group discussions (FGD) organized in 41 villages and 7 sectors of Nouna’s town to discuss criteria and perceptions of poverty. The discussions were audio recorded, transcribed and analyzed in French using the software NVivo 9.

**Results:**

From the FGD on poverty and the subjective definitions and perceptions of the community members, we found that poverty was mainly seen as scarcity of basic needs, vulnerability, deprivation of capacities, powerlessness, voicelessness, indecent living conditions, and absence of social capital and community networks for support in times of need. Criteria and poverty groups as described by community members can be used to identify poor who can then be targeted for subsidies.

**Conclusion:**

Policies targeting the poorest require the establishment of effective selection strategies. These policies are well-conditioned by proper identification of the poor people. Community perceptions and criteria of poverty are grounded in reality, to better appreciate the issue. It is crucial to take these perceptions into account in undertaking community development actions which target the poor. For most community-based health insurance schemes with limited financial resources, using a community-based definition of poverty in the targeting of the poorest might be a less costly alternative.

**Electronic supplementary material:**

The online version of this article (doi:10.1186/s12889-014-1335-4) contains supplementary material, which is available to authorized users.

## Background

The fight against poverty remains a universal concern [1-4]. In most developing countries, poverty issues are mainly addressed with regard to the implementation of national strategic plans for poverty alleviation [5-9]. In the 1970s, definitions of poverty focused mainly on income [10-14] and were often based on a comparison of individuals’ income and consumption with some defined thresholds, below which individuals were considered poor [15,16]. New layers of complexity in the definition of poverty were added in the 1980s, including some non-monetary aspects [17-21]. Poverty is associated not only with insufficient income or weak consumption, but also with insufficient outcomes in respect to health, nutrition, literacy, deficient social relations, insecurity, low self-esteem and powerlessness [22-29].

Increasingly, a qualitative approach to an understanding of poverty is emerging, involving the cultural and socio-economic aspects, along with the individual perceptions of population [30-33]. The complexity of measuring poverty mirrors the plural character of its definition [21,25-29,34,35]. This complexity increases where participatory methods are used and framed in building on the indicators of poverty defined by the target population [36-41]. Indeed in the 1990s, emphasis was put on how poor people themselves viewed their situation [20,39,42]. The local population that lives and works in the same community is in a position to observe the economic status of fellow community members over a long period and can be considered to be a better judge to assess levels of wealth [43-46].

It is now universally recognized that poverty is a complex and multidimensional issue [17,21,25-27,29,34,35,47]. It is generally defined in relation to a given context, which may be global, regional, national, local or individual [48-52]. It has become the norm not to limit research in that area by considering only the monetary aspects of poverty [35].

In the context of scarcity of resources, support for disadvantaged groups requires that the poor individuals are identified correctly and that their needs are well understood [20,53-55]. Acknowledging the ways a given community defines poverty may be critical in how members of the community choose coping strategies [56-58]. Community-based definitions of poverty were required in various rural settings to target poor people and to address poverty issues. Indeed, Rai showed how Governments and aid agencies could reduce the costs in targeting the poor by using community information [39]. Community concepts of poverty have been used to provide premium exemption in the Ghana National Health Insurance Scheme [46]. A community wealth ranking was performed in Nouna, Burkina Faso, to target poor households for enrolment subsidies [40]. Collins, in Niagara Falls, Canada, tried to understand poverty from those who are poor [55]. A series of researches put emphasis on the voices of the poor in addressing poverty issues [59-62]. In Indonesia, the poor were targeted using three approaches, among them community wealth ranking [63] Community Strategic Visioning was used as a method to define and address poverty in rural Montana [64].

In 2004, a community-based health insurance (CBHI) was launched in the Nouna’s health district in Burkina Faso with the main purpose of improving access to health care. The enrolment was at the household level and households were required to renew their membership yearly [65-68]. Enrolment among the poor households was very low throughout and financial barriers were mentioned as the main reason for not enrolling in the scheme [40,69]. In response to this, in 2006, the CBHI steering committee decided to provide premium subsidies to poor households [40]. In 2007, a method of self-assessment named community wealth ranking (CWR) was used to identify the poor households in each community [40,70-72].

The CWR’s method briefly entailed three sequential steps: First, a focus-group discussion (FGD) with knowledgeable people within the community was conducted to understand the key local criteria or characteristics of poverty and then to apply these criteria to identify main poverty groups in the community. Second, the households ranking was conducted. In this exercise, cards with the names of household heads (HHH) were divided into three different color piles. This ranking of households was performed separately by 3 key-informants, each one with a pile of card. These key informants were selected by the participants of the FGD as being knowledgeable of the socio-economic status of each HHH in the village or the Nouna’s sub-sector. In the last step, consensus among the key informants was reached. Three types of consensus were noted: Either there was an agreement in the rankings of households, or a pairwise agreement; or again, the HHHs were selected by only one of the three key-informants. In that last case, the names of HHH were reviewed together by all the three key-informants, to ensure that they all agreed on their assigned rank. The list of selected HHHs for subsidies was elaborated and announced to the participants in the FGD [40,72].

The consensual community-based definition of poverty was the key-element used by each key-informant to classify the HHH in the predetermined poverty groups. The useful contribution of the community allowed the steering committee of the CBHI scheme to use this method to identify poor households for premium subsidies to enroll in CBHI.

This paper aims to provide a new set of poverty criteria, based on community perception, which could be used to build systematic indicators for surveys aimed at measuring and alleviating poverty in the Nouna region.

Targeting the poor using a community-based definition of poverty, as stated in the paper, could also be useful for most community health insurances in the region with limited financial resources. Finally, the paper attempts to contribute to the debate in the research area about how poverty should be defined. The alternative definitions of poverty provided by community members, that are not in purely financial terms, are highlighted. The paper presents the definition, criteria and poverty groups defined by community members for targeting the poor in Nouna’s health district.

## Methods

This is a descriptive and exploratory study using qualitative methods.

### Study area

Our study took place in 2009, in 41 villages and 7 sectors of Nouna’s town, located in Nouna’s Health Demographic Surveillance Site (NHDSS) with an area of 1756,244 km2. It should be noted that Nouna’s town is a semi-rural setting [73]. The NHDSS was established in Nouna’s Health District, which is one of the six districts of the health region of Mouhoun. The region is a dry orchard savannah and has a sub-Sahelian climate with a mean annual rainfall of 796 mm (range 483–1,083 mm) over the past five decades [73]. It covers the administrative boundaries of the province of Kossi with an area of 7464.44 km^2^. The NHDSS has 34 peripheral health facilities (CSPS) and a district hospital (CMA). The mostly rural population of the multi-ethnic Kossi’s province consists predominantly of subsistence farmers and cattle keepers [73]. The dilapidated road network made economic activity difficult throughout the province (see Additional file 1).

### Study population

The study target population consisted of all the HHH regardless of gender or membership status to the insurance scheme in the 41 villages and 7 sectors of Nouna’s town where CBHI was implemented. The household was defined as the socio-economic unit in which individuals live together, share resources and jointly satisfy their needs under the authority of a HHH [74]. The database of NHDSS was used to identify all the 7,807 heads of household in 2009, in the settlement area of CBHI. The main ethnic groups in the study setting were the Marka (38%), Bwaba (25%), Mossi (18%), Fulani (8%) and Samo (7%) [73]. The Dioula language serves as a lingua franca, permitting communication between the different ethnic groups in this region [73]. In terms of religious beliefs, the study population consists of Muslims, Catholics and Protestants.

### Method

Focus Group Discussions (FGD) were conducted in 41 villages and 24 sub-sectors of Nouna’s town, totaling 65 FGDs. Seven sectors of Nouna’s town were divided into sub-sectors due to the large number of households in these sectors.

- Size and composition of participants for the FGD

The number of participants in FGD met the following premises: a small group (less than 4 participants) could be less productive because participants are more sensitive to the dynamic exchanges between them. On the other hand, a larger group (more than 12 participants) could be difficult to control with a high risk for participants to become shy, leading to the creation of sub-groups and to the deterioration of the conversation [75,76]. Thus, the FGD size was between 4 and 12 participants. On average, we had 10 participants per FGD.

The FGD participants had lived long enough in the community and had a good knowledge of the socio-economic status of each household. They could be opinion leaders like the chief of the village, religious leaders, elders, etc. They could also be leaders of associations or groups like farming cooperative, artisan groups and associations of women. The FGD participants included both men and women regardless of age, ethnicity and religion. The women in the NHDSS have previously been involved in mixed FGD, where both men and women participate, and therefore, were comfortable voicing their opinions in such discussions. Women represented about 1/3 of the participants in our FGD.

b) Information strategies

The Social Mobilization Unit of the CBHI, operating at different levels (central and local), informed the community members about the community wealth ranking exercise and gave information about the venue and time when it would be undertaken in the village. Community members were also informed via local radio networks and door-to-door information was conveyed by local contacts of the CBHI scheme. When appropriate, drummers were used for announcing the meetings in the villages.

c) Running of FGD:

The field workers in NHDSS knew the two main spoken languages (Dioula and Bwamu) in the study setting. They had extensive experience in conducting FGD in this region. After a consensus among the participants on the language, the principal investigator and field workers facilitated the discussions. The discussions were mainly conducted in Dioula, but we noticed that some participants felt more comfortable in giving their point of view in Bwamu, which is the second main spoken language.

The participants were asked to discuss their ideas on poverty – how they define poverty, criteria they use to differentiate poverty groups and the terms they use for describing poverty. Exchanges conducted with participants on various topics related to poverty were consensus-based. The main criteria provided by the participants to define poverty were listed and presented to them in order to get their consensus.

d) Data analysis:

The discussions during the FGDs were audio recorded and then translated and transcribed into French by transcribing agents knowledgeable in the two main local languages in which the FGDs were held. The transcripts were then typed by the Data Entry Unit of CRSN.

The Content analysis, aiming to categorize verbal data for the purpose of classification, summarization and tabulation, was used to account for what was said by participants in the most possible objective and reliable way. Two levels were considered for the content analysis, one was descriptive about the data and the other interpretative about what the data meant.

After reading the transcripts, semantic units, or themes, were extracted, based on the frequency of use by participants to express an idea or opinion. These themes were subsequently classified into categories, or containers, related to the objectives of the study. In our study, the categories were consistent with the poverty criteria as defined by the community members.

The manual coding method was used to label data in Nvivo. First, some emerging themes in terms of the frequency of use were put into containers called free nodes. Then, from the text, the related recurrent themes were dragged and dropped into the nodes. Some free nodes, therefore, were grouped into tree nodes according to the research objectives. Some direct, representative and relevant quotations were used verbatim within the text to enlighten emerging themes. The software NVivo 9.2 (QSR property limited. From 1999 to 2011) was used for this qualitative analysis. Analysis was done by the principal investigator.

e) Ethical approval

The study protocol was approved by the institutional Research Ethics Committee of CRSN (2005–005/CLE/CRSN) and as a component of D2 project by the ethical committee of the University of Heidelberg (Medical school) under the number (130/2002). FGD participants’ consent was obtained before the start of each FGD. Emphasis was on the confidentiality of data within each village, in order to avoid information leaking from one village to another, and also to avoid disclosing information about poorest households.

## Results

In general, poverty was seen in this study area as scarcity of basic needs, vulnerability, deprivation of capacities, powerlessness, voicelessness, indecent living conditions, and absence of social capital and community networks for support in times of need. The definition of poverty was mostly associated to the geographical, social and cultural contexts of daily life by the participants. A HHH and farmer in the village of Barakuy reported: ‘The wealthy in one village may be considered poor if we change the benchmark’ (Male, HHH, 41 years old). This assertion was also raised by several participants in many other villages. The complexity of poverty issues was also raised when we tried to define and identify those poor or wealthy. The village’s informer in Bourasso who participated in many activities and studies implemented in the village stated ‘In a meeting if you ask the poor to raise their hand, no one will do it. In the meantime if you direct the question to the wealthy ones, the behaviour will be the same’ (Male, 48 years old).

### Local names and meanings of poverty

The word poverty, when translated into local languages, carried different connotations. In their local languages, communities used words that were full of meaning in their ways of designating poor and rich people. The names provided to point out a poor person were basically a description of the situation or the behavior of that person. According to the various ethnic groups, the commonly used names and meanings given to poor people are listed in Table 1.

**Table 1:** **Local names full of meanings of poverty**

**Local languages**	**Local names of the poor**	**Translation into English « literal » and «** ***meanings*** **»**	**Local name of the wealthy**	**Translation into English « literal » et «** ***meanings*** **»**
Dioula	Dêssêbagato	«dêssê = has tried in vain/ bagato = person » *« the one who is not able, who has not succeed, who has failed»*	Nafolotigui	« nafolo = wealth/tigui = owner »
				*« The one who has wealth, who has the fortune »*
	Faantan (word from Faangatan)	« faanga = authority, power/ tan = lack »	Faanman (word from Faangaman)	«faanga = authority, power/ man = owner » *« the one who is leader, who has power and wealth*»
		*« The one who is powerless, who has nothing»*		
	Tiguèlankoro	«tiguê = hands/ lankoro = empty*»*	Séétigui	« sée = capability, power/ tigui = owner » « the one who is able, who has power*»*
		*« the one who owns nothing »*		
			Fintigui	« fin = thing/ tigui = owner » *« the owner, the one who has wealth»*
			waritigui	« wari = money/ tigui = owner»
				*« the one who has money, the wealthy »*
Marka	Paantan (word from Paangatan)	« paanga = authority, power/ tan = lack »	Paaman (word from Paangaman)	«paanga = authority,power/man = owner » *« the one who is leader, who has power and wealth »*
		*« the one who owns nothing, who is a needy»*		
			Pinti	« pin = thing/ ti = owner » *« the owner »*
Moré	Nongsoaba	« nongo = tribulation/ soaba = owner » « the one who,suffer, who is a needy»	Raakanre(word from Rahawakanre)	«kanre = prosperity/ rahawa = the man » *« the one who thrives, who has happiness»*
	Taalga	« taalga = a needy» *« the one who has nothing, who has to beg to survive »*	Liguidisoaba	«liguidi = money/ soaba = owner »
				*« the one who has money, who is wealthy »*
			Arzeksoaba	« arzeka = fortune, prosperity/ soaba = owner » *« the one who thrives, who can afford »*
Bwamu	Baabasso	« baaba = needy/ so = owner » « *the one who is a needy, who has to beg to survive »*	Séébasso	« sééba = capability, power/so = owner » « the one who is able »
	Fioro	« fio = needy/ ro = owner »	Biobasso	«bioba = thing/ so = owner »
				*« the owner, the one who has wealth »*
		*« the one who is a needy, who has to beg to survive »*		
Fulani	Taalka	« taalga = needy»	Djomdjawdi	« djawdi = wealth/djom = owner »
		*« the one who has nothing, who is a needy »*		*« the one who has wealth, who has power»*
	Missikina	« Missi = beef/kina = lack » « the one who doesn’t own beef »	Djomnadjê	« nadjê = beef/djom = owner »
				*« the one who owns beefs »*
	Nyaagoodo	« Nyaagaade = needy/ do = owner » *« the one who is a needy »*		
Samo	Douoba (word from Dounbamba)	« doun = will/bamba = non achieved » *« the one who cannot solve his own problem without any support»*	Padina	« Pa = money/ dina = owner »
				*« the one who has money, who is wealthy»*

### Number and types of poverty groups identified

The participants in the FGDs were asked to identify the numbers and types of poverty groups in their villages or Nouna’s sub-sectors. The results showed that 61 (94%) FGDs identified mainly three poverty groups: poor, intermediates and wealthy households. Only 3 (5%) FGDs referred to two poverty groups: poor and wealthy; and 1 FGD listed four types of poverty groups: very poor, poor, intermediate and wealthy.

### Relative poverty criteria for each poverty group

The links between the identified poverty groups and the related criteria is summarized in Table 2.

**Table 2:** **Relative poverty criteria for each poverty group**

**Poverty groups**	**Very poor**	**Poor**	**Intermediate**	**Wealthy**
**Criteria**				
Has insufficient food	++	+		
Has nothing	++	+		
Is not in good health status preventing to work	++	+		
Is unable to solve his own problem by himself	++	+		
Has no money	++	+		
Is unable fill his medical prescriptions	++	+		
Has no social network	++	+		
Is not owner of livestock	++	+		
Wear poor clothes and live in poor conditions	++	+		
Is very old and without support	++	+		
Has no poultry	++	+		
Has enough food			+	++
Can do or get anything			+	++
Is in good health status			+	++
Is able to solve his own problem and help somebody else			+	++
Has enough money			+	++
Is owner of livestock			+	++
Has nice clothes and good living conditions			+	++
Can use health services			+	++

+: The cross indicates that a link exists between the criterion and the poverty group.

++: The two crosses indicate that a strong link exists between the criterion and the poverty group.

The shaded area: indicates that the criterion is not related to the poverty group.

#### Poor group

The criteria that defined a person as poor were mainly: Has insufficient food, has nothing, is not in good health status preventing work, is unable to solve his own problems by himself. Besides this main group of poverty criteria, some others were also mentioned by community members to refer to the poor: has no money, is unable to fill his medical prescriptions, has no social network, is not an owner of livestock, wears poor clothes and lives in poor conditions, is very old and without any support, and has no poultry. The FGD’s participants decided that anyone who fulfils at least one of the poverty criteria mentioned by them is defined as ‘poor’.

**Figure 1:**
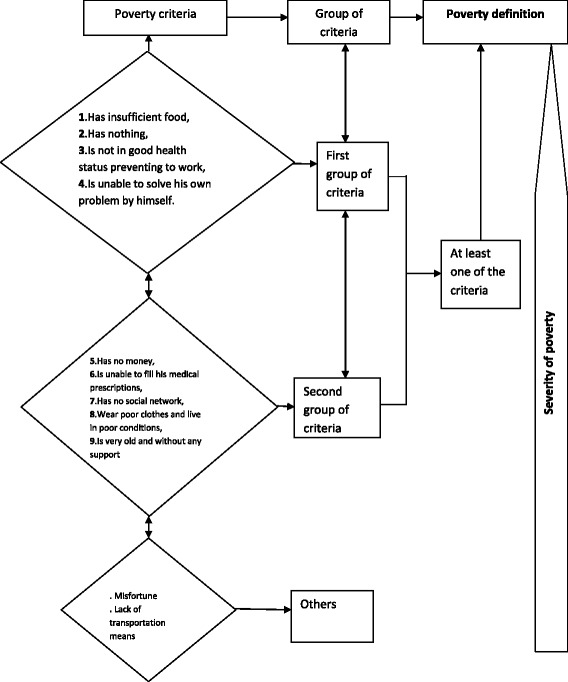
**Algorithm of the definition of poverty by community in Nouna, Burkina Faso.** →: contributes to. ↔: are interconnected.

All these criteria contributed to making the poor powerlessness, voicelessness and vulnerable. Some criteria were classified as “others” and were related to the misfortune, the lack of means of transportation and the disappointment of the poor people. The above information were translated into an algorithm to define poverty in the study area (see Figure 1).

Not having a sufficient daily meal for a household was identified as the criterion which mainly emerged from the discussions with the participants to designate the poor. This was stated by many participants, among them a farmer in the village of Boune who said: ‘The poor is the one who cannot get his daily meal and eat his fill’ (Farmer, male, 54 years old). Beside insufficient food, the absence of ownership, translated into having nothing by community members, was mentioned several times. Poverty was also related to the poor health status of the household’s head. Indeed, in order to perform their farming activities for most of them, the HHHs needed to be in good physical and mental health. Otherwise, they were considered poor, as pointed out by a farmer in the village of Bourasso: ‘The poor is the one who is lying down, who cannot do anything and who cannot even get up to work’ (Farmer, male, 49 years old). The poor were also seen as unable to solve their own problems, making them dependent on the community. A female HHH and farmer in the Village of Dara raised the fact that the poor had no point of view in the main decisions of the village, otherwise, even if they gave their opinion, it was not considered: ‘When you are poor, you are not considered, nobody wants to invite you for the meetings in the village; you rather stay at home because they will never consider what you would have said even if you were there’ (Female, HHH, 37 years old).

**Figure 2:**
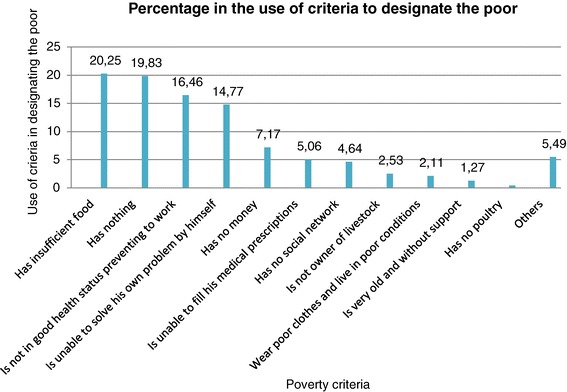
**Percentage in the use of criteria to define poverty.**

The percentage in the use of these criteria by community members to designate the poor during the 65 FGDs are showed by Figure 2.

#### Intermediate group

The intermediate HHH were those who had their daily meal, those who did not need to beg, in respect to food, materials of agricultural work, means of travel, etc. This group is characterized by those who have sufficient means for themselves, but may not be able to help needy ones: This was illustrated by a male, local head of CBHI in the village of Labarani: ‘People in intermediate group can get their daily food; they are not considered poor, but they are not however considered rich’ (Male, 39 years old).

#### Wealthy group

The last group is composed of the wealthy households that are characterized by the opposite of all that was cited for the preceding groups.

From the debates with community members, it came up that the wealthy fulfilled at least one of the following wealth criteria: is able to solve his own problem and help someone else, has enough money, is owner of livestock, has enough food, can do or get anything.

Besides the main criteria, the wealthy were also designated by another category of criteria: has enough food, can do or get anything, is in good health status, wear nice clothes and good life condition, can use health services.

Some criteria of wealth classified as “others” referred to: Has modern transportation means like motorcycle and car and has built a house in block. Wealthy people are self-sufficient in their life. They are able to cope with their own problems and those from needy ones, said a housewife in Lei’s Village. ‘In our village, if you see someone who can solve his own problem and additionally can support poor is called wealthy’ (Female, 35 years old).

‘The livestock are part of the wealth, since money is not a tree that can be planted and reap the fruit. These are things that we can barter or we sell them to get enough money and become wealthy’ (Male, 43 years old). This assertion, which highlights the ownership of livestock as evidence of wealth, was from a male and HHH who was both a farmer and stockbreeder in Diamasso’s village.

Likewise, community members put great emphasis on the importance of health in their everyday life as noted a farmer and chief of Solimana village. ‘The wealthy is the one who is healthy since health is the most important wealth’ (Male, 61 years old). They also focused on school knowledge and monthly wages for those in the sectors of Nouna town to designate the wealthy. A jobless man without any school degree, asserted: ‘For us, someone who can be called wealthy is the one who has monthly pay, who has some school knowledge’ (Male, 36 years old).

**Figure 3:**
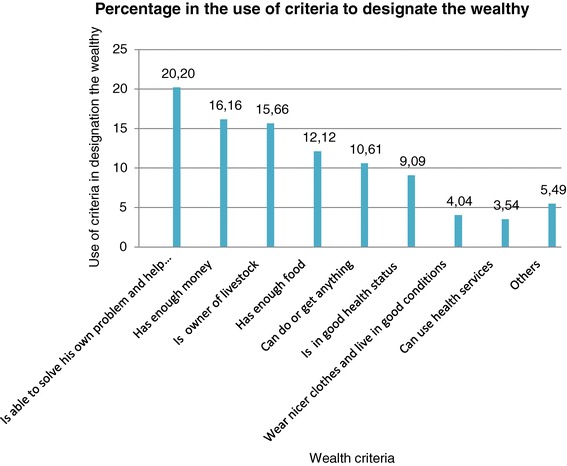
**Percentage in the use of criteria to define wealthy.**

The following Figure 3 showed the percentage in the use of these criteria by community members to designate the wealthy during the 65 FGDs implemented.

### Relative poverty criteria for each poverty group

When we applied the local definition of poverty provided by the community members, the groups most exposed to poverty were identified. Although community members believed that poverty makes no difference and could affect anybody in general, they recognized, nevertheless, that poverty tended to affect mainly the elders, followed by the ill and disabled people who could no longer work, the women, and finally orphans and children without support.

## Discussion

Discussing poverty issues in a community is a very sensitive subject and needs to be approached with great caution. Mckendrick stated, in the case of UK, that talking about poverty is never neutral and the language may be sexist, ageist, racist or homophobic if not used appropriately [77,78]. Some other authors also noted the sensitive nature of poverty [79-82].

Community members in Nouna identified mainly three poverty groups: poor, intermediates and wealthy. We noted that only one FGD indicated 4 poverty groups including the very poor. But on the other side, no FGD mentioned the wealthiest group as a poverty group to be considered since they rejected the fact that there were wealthiest people among them. We also noted that these community members did not make any great difference between the poor and the very poor. For them the two groups faced the same difficulties with only slight differences. The Food and Agriculture Organization of the United Nations (FAO), in a study implemented in the Horn of Africa, also found three poverty groups: the lowest levels of Wealth Index, the intermediate Wealth Index and the high Wealth Index [83]. Kalisa in Rwanda studied the characterization of farming systems in southern Rwanda and found three wealth categories among the farmers: Well-off farmer, Intermediate farmer and the Poor farmer [84].

The community-based definition of poverty that we used in our study allowed us to better understand poverty from the view of the community. Souares, in Nouna, targeted the poor households for insurance enrolment subsidies by using a community-based definition of poverty [40]. The same exercise was performed in Ghana by Aryeetey who also used the community concepts of poverty to provide premium exemptions in Ghana National Health insurance scheme [46]. Many other authors have also used this community-based definition of poverty to target the poor [39,55,63,80].

Making community central to the definition of perceived poverty was guided by the fact that community members might have a better feeling for poverty and might understand all the arguments for that. This was also mentioned by some other authors [43,46,85,86].

We were convinced that the poverty criteria provided by community members reflected a realistic situation experienced by them. They were the ones concerned with poverty issues, so they were the ones in the best position to express what they felt in their own words and languages.

Most of the criteria highlighted in our study referring to the poor, like the insufficient daily meal, the ill-health …etc., were also mentioned in the study of Aryeetey in Ghana. ‘The poorest were unable to afford three meals daily and meals were often made up of cooked cassava…’[46]. She also indicated, as we mentioned in our study, that it was common to see the poorest wearing miss-matched old plastic bathroom slippers and worn out, often dirty clothes.

In our study we pointed out that poor people were sometimes excluded from the main decision making because of their poverty status. This situation was also noticed by Aryeetey in Ghana. ‘The poorest people were usually not included in group decision making processes either at the family or community level’ [46].

Our study showed, as did several other authors, that involving community members in the definition of poverty may allow poor people to make their voices heard [20,59,60,86-90], as they are often not considered in their own society. This situation of marginalization was highlighted in 2010 by Salmen [91,92]. Narayan in 2000 [54] recognized that economically marginalized groups also tend to be socially marginalized. In Kenya, Cameroon, Gabon and Zambia, he reported previously that poor people felt powerless and that they were generally voiceless [93].

In the 41 villages where our study took place, the concepts of monetary incomes and expenses remain little utilized. So using community concepts to define poverty was more informative for our study than using a monetary assessment.

The same observation was made by Saul in 1999. The community’s way of defining poverty is, thus, very important and quite relevant, in so far as the main determinants of formal poverty assessment, such as incomes and expenses, are not generally well-known in monetary terms in this rural context [94]. Coudouel also found in her research that measuring intrahousehold allocations and inequality was difficult, since the available data typically failed to directly capture individual spending and consumption [95].

Alatas found in Indonesia that the results of community-based methods were more strongly correlated with individual community members’ self-assessments of their own status than a metric of poverty based purely on consumption [63]. This has also been highlighted by Chambers and Salmen [37,92].

Our study also highlighted, as Chambers did, some aspects of poverty referring to “vulnerability and powerlessness”, which are not captured in conventional surveys [36,37,96,97]. The scarcity of basic needs as referring to the poor was also indicated by many authors [7,91,98-100].

We might assert about the process aimed at targeting the poor, that using the understanding of “who was poor” and “what were the defined poverty groups” provided by community members as key-elements for the selection of those poor, was less costly in terms of time and resources. Chambers, in 1994, published that this community-based method appeared to be an important cost-effective alternative to the normal surveys, particularly with regard to the time and budget used for the surveys [37].

We would like to mention three implications regarding our research. The first implication is the important role and place of community members in defining ‘perceived’ poverty and towards poverty alleviation in Nouna health district. Poverty was mainly defined by community members outside traditional economic descriptions, this should lead policies in Burkina Faso to put greater emphasis on the contribution of Community in defining poverty and targeting poor for poverty alleviation. The contribution of the paper to the debate about how poverty should be defined, might then be considered. The second implication is that, using community-based definitions of poverty in targeting the poor for subsidies or exemption within a community, may be cost-effective for organizations, like community-based health insurances, with limited financial resources. The third implication is that our research provides a new set of poverty criteria as seen and perceived by the community that could be used to build systematic indicators for surveys aiming to measure and alleviate poverty in the Nouna region. Indeed, Burkina Faso in its Strategy for Accelerated Growth and Sustainable Development (SCADD) from 2011 through 2015, which replaced the Poverty Reduction Strategy Paper, aims to alleviate extreme poverty and hunger as specific objective number 2 [101]. Our research could help Burkina Faso policy-makers better target the poor and more effectively address poverty issues in community areas.

We are confident that any policy or program addressing poverty issues in a specific community should consider how that specific community defines poverty.

Some limitations of our study should be mentioned, since we made the choice of inviting knowledgeable people within the community to discuss on its behalf. It was certain that the participants invited to take part in the discussions for the definition of poverty and poverty groups gave their own perceptions and concepts, which might be identical or slightly different from the general community view. Also, the study listed some relevant criteria referring to poverty within the community, without building systematic indicators for a poverty survey. Some nuances between poverty groups ‘poor vs. very poor’ and ‘intermediate vs. wealthy’ were not easy to define by community members.

## Conclusion

The definition of poverty by community members to be used in the targeting of the poor, seems to be one of the most adequate ways in a setting where people live in a community, sharing daily realities, like in Nouna’s health district. For most community-based health insurance schemes with limited financial resources, and particularly the CBHI in Nouna, using a community-based definition of poverty in the targeting of the poorest might be a less costly alternative.

Our study allowed us to elicit a new set of criteria that could be used to build systematic indicators for surveys aiming to measure and alleviate poverty in the Nouna region. This research could help Burkina Faso policy-makers better target the poor and more effectively address poverty issues in community areas, by taking into account the perceptions and criteria of poverty as seen and defined by these communities in addition to applying the unified questionnaire on basic welfare indicators already in use in the country.

In the context of establishing subsidies for the poor in a particular community, the involvement of this community in defining who is eligible and the targeting of these eligible people may appear to be an essential element for the acceptance of the potential subsidies.

Indeed the targeting of the poor in Nouna’s health district, using community definition of poverty, aimed to provide insurance premium subsidies to those selected as poor.

We assert that policies aiming at poverty aleviation in a given community, should consider the contribution of that community in defining and aleviating poverty. Our study attempts to contribute to the debate in area of research about how poverty should be defined.

## Additional file

Additional file 1:
**MAP of Nouna Health Demographic Surveillance Site.**

## Abbreviations

CBHI: Community-based health insurance
CLE: Comité Local d’Ethique
CMA: Centre Médical avec Antenne chirurgicale
CSPS: Centre de Santé er de Promotion Sociale
CRSN: Centre de Recherche en Santé de Nouna
CWR: Community wealth ranking
FAO: Food and agriculture organization of the United Nations
FGD: Focus group discussion
HHH: Head of household
IMF: International monetary fund
MAED: Ministère des Affaires Economiques et du Développement (Niger)
MED: Ministère de l’Economie et du Développement (Burkina Faso)
NHDSS: Nouna health demographic surveillance site
QSR: Qualitative solutions and research
SCADD: Strategy for accelerated growth and sustainable development
UN: United Nations
UNDP: United Nations development programme
VS: Versus
