# Supplementary figures and images for: Using a community-based definition of poverty for targeting poor households for premium subsidies in the context of a community health insurance in Burkina Faso
Source: BMC Public Health. 2015 Feb 6;15:84. doi: 10.1186/s12889-014-1335-4 (PMC4337311; doi:10.1186/s12889-014-1335-4)

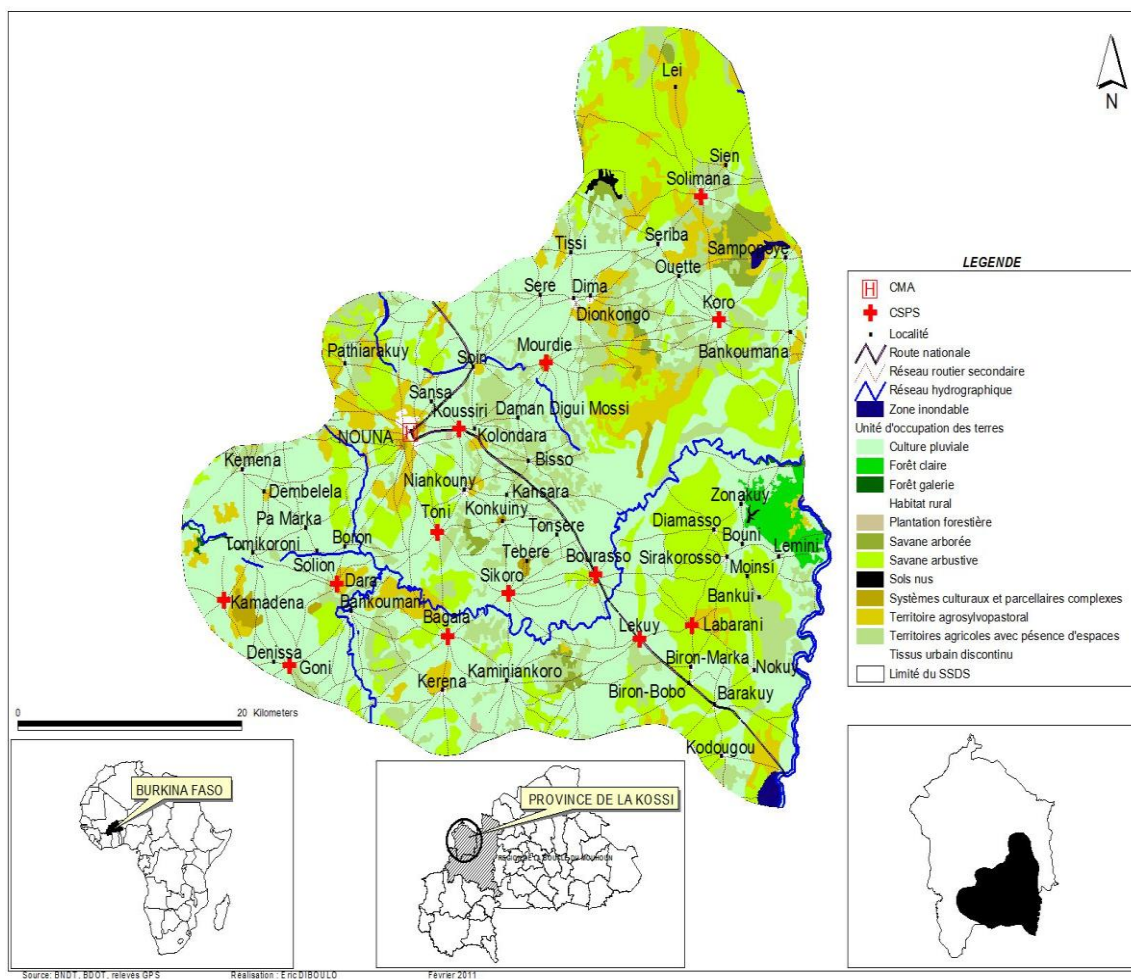

Supplement: Additional file 1: — MAP of Nouna Health Demographic Surveillance Site. [file 12889_2014_1335_MOESM1_ESM.pdf]
